# Everybody's Page

**Published:** 1888-01-21

**Authors:** 


### Everybody's Page.

"Perhaps now that you have heard how the cabman spends his time while we are dancing, you will sometimes think of him too," is a remark which Leslie Keith, in a story not long since concluded in our pages, puts into the mouth of her heroine. It is to be feared that, as a rule, neither host nor guests remember coachmen and cabmen waiting outside in the raw night air, till their employers shall think fit to come away. Even on this matter, however, the public conscience is awaking, as the recent action of a lady at Kensington shows. She arranged that on the evening on which she gave a dance, the keeper of a coffee-stall should take up his station opposite her house, and distribute coffee, cocoa, and slices of currant cake to her guests' coachmen. About eighty quarts of coffee and cocoa were drunk during the night, and a proportionate quantity of cake was consumed, the total cost being about thirty shillings, not in itself a very large sum to add to the expenses of a dance—it could be saved in flowers, if necessary, without any one being much the worse —and the gratitude such an evidence of consideration for their comfort would win from the drivers themselves should make our hostesses think it well expended

"Is marriage going out of fashion?" asks the *Pall Mall Gazette* in a voice of anxiety. "Our present civilization demands a standing army of women," says the *Daily News*, and clearly gives its readers to understand that the modern female battalion, like the Amazons of old, despise matrimony and its joys and cares. The old proverb says that "when the gorse is out of bloom, kissing 's out of fashion;" and we think that marriage will go out of fashion about the same time—that is, in the Greek Kalends. But it is certainly true that marriage is no longer what Mr. Haweis once called it—"the only profession open to the great majority of women." Women do not now regard marriage as a profession, but as a privilege, which may or may not be offered to them in a fashion they care to accept. This feeling, which is a direct result of the increasingly active part women are taking in the work of the world, certainly lessens the number of marriages while raising the ideal of it to a higher level; and women, having accepted celibacy as their lot in youth, are now making provision for their old age. The spinsters of a certain German town have formed a friendly society, the members of which are by payment of a small premium assured of an allowance in sickness and a pension in old age. Every member who marries forfeits thereby all claim to the benefits of the society; and doubtless it is from the apostasy of these tender-hearted renegades that the society expects to make its profits. The scheme is a good and sensible one, and disproves the common assertion that women can't co-operate; but—the neighbouring bachelors don't like it. They feel that the consciousness of being provided for in old age will make the maids of their choice more coy and hard to win than ever; and apparently, poor fellows! they would rather be accepted for a poor reason than refused altogether. Therefore, in spite and emulation, they have set up a rival institution—"The Bachelors' Mutual Aid Society." The nature of its provisions have not yet been made public; but doubtless the intention is to bring sorrow and remorse to the hearts of the rebellious spinsters.

One of the trials—the many trials—of the young author who wishes to take the public by storm, is the difficulty of finding new and original incidents which can bear the analysis of the sceptical critic. If he does not shoot his villain or make him die in the agonies of poisoning by strychnine—elaborately, even if inaccurately, described—or otherwise dispose of him in the conventional way, he is told that he is going beyond the bounds of that which is conceivably possible, even in fiction. The experienced hand does not care for this; indeed Mr. James Payn boasts that a few years after he had invented a very original—so-called impossible—*dénouement* to his plot, Nature herself did him the honour to copy it, thereby reducing it to the possible—to the plains of fact from the mountain peaks of imagination. But as everyone has not the courage to give Nature a lead, so to speak, we offer the inexperienced novelist the history of a case that lately came before the East London coroner. A commercial traveller named Moses Raphael, of 100, St. Paul's Road, Bow, was taken ill, and on Monday week was removed to the London Hospital. His death took place on Tuesday, and at the inquest held afterwards, Dr. Doyle, who had had the man under his care, said that on opening the head he found a penholder and nib about three inches long attached to the right orbital plate. It must have been there for a considerable time, as the bone had partially grown over it. The only way that it was possible for the pen and holder to get to the brain was by passing through the eye or up the nostrils. Deceased's widow stated that her husband had never complained of any accident, but that lately he had suffered from pains in his head. Dr. Doyle said it was a mystery how a pen and holder of such a size could get into the brain without the man's knowledge. This case will probably remain for a long time an unsolved puzzle to surgeons and anatomists, and we venture on no explanation of the strange accident. But after a fairly complete study of the works of Messrs. Haggard and Stevenson we think that fact still surpasses fiction in strange and incredible incidents.

The time when ballooning shall have become a popular and well-regulated means of locomotion has often been prophesied, but has not yet arrived, nor does its advent seem hastened by an accident which occurred a short time ago, or is, one may say, occurring now. It is, however, some weeks since M. L'Hoste and a companion—M. Margot —ascended in a balloon from the coast of America, and nothing has been heard of them or their machine since. Whether they have ascended to a region whose atmosphere is so rarefied that human beings cannot exist in it, or whether in the recent stormy weather they have been blown out to sea, have descended upon the Atlantic and been drowned, no man can tell; but their experiment proves that the "voyage en ballon," while it may be a suitable subject for Jules Verne's romances, and even, under careful restrictions, for the scientific amusement of the curious, is not yet a method of travelling for reasonable people to adopt.

The Americans have something new to teach us. They have established hotels for the street Arabs of New York, the boys who earn their living by selling matches, newspapers, etc. The cost of a bed at these caravanserais is five cents (2½*d.*); supper and breakfast of coffee and bread and butter, each five cents. The only condition attached to the use of the hotels is that each guest shall indulge in a thorough wash when he comes in, and that he must arrive before midnight. The hotel possesses a covered playground, a reading-room, provided with story-books, illustrated papers, dominoes, draughts, and other games. There are also classes held in the evening, which any boy may attend gratis. There is a piano in one room, and concerts and conjuring entertainments are frequently given. If, however, a boy wishes to go out to theatre or music-hall, he is permitted to do so; no restriction is placed on his liberty. Besides these advantages, occasional "surprise-breakfasts"—the addition of bacon or cold meat to the regulation bread and butter—have made the institutions very popular. Altogether, the New York Arabs seem to be exceptionally fortunate, and as the institutions are to a great extent self-supporting, there is no reason why similar ones should not be started on this side of the Atlantic.

